# Estimation of cutoff score for the 7C of vaccination readiness scale

**DOI:** 10.1016/j.jvacx.2023.100394

**Published:** 2023-09-26

**Authors:** Masaki Machida, Tomoko Takamiya, Yuko Odagiri, Noritoshi Fukushima, Hiroyuki Kikuchi, Shigeru Inoue

**Affiliations:** a Department of Preventive Medicine and Public Health, Tokyo Medical University, 6-1-1 Shinjuku, Shinjuku-ku, Tokyo 160-8402, Japan; b Department of Infection Prevention and Control, Tokyo Medical University Hospital, 6-7-1 Nishishinjuku, Shinjuku-ku, Tokyo 160-0023, Japan

**Keywords:** Vaccine hesitancy, Vaccine acceptance, Measurement, Questionnaire, Cutoff score

## Abstract

The 7C of the vaccination readiness scale provides a score to evaluate the willingness and readiness to get vaccinated (vaccination readiness). This cross-sectional study clarifies the cutoff vaccination readiness score to determine whether an individual is willing to be vaccinated. We conducted an online survey of 774 participants. The vaccination readiness scores for the full and short survey versions were calculated. The coronavirus disease 2019 vaccination status was used to determine whether the participant was willing to be vaccinated. Receiver operating characteristic curve analysis was performed using the Youden index. The Youden Index was at its maximum when the cutoff score was 4.02 for the full version (area under the curve [AUC]:0.94) and 4.07 for the short version (AUC:0.90). Vaccination readiness scores greater than 4 on both the full and short version may indicate a willingness to be vaccinated. This cutoff value facilitates the interpretation of the 7C scale results.

## Highlights

• Cutoff score for the 7C of the vaccination readiness scale was clarified.
• Receiver operating characteristic analysis calculated full and short survey scores.
• Vaccination readiness score over 4 indicates a willingness to be vaccinated.

## 1 Introduction

Vaccine hesitancy has become a global public health concern [1]. Various scales have been developed to evaluate thoughts and intentions regarding vaccines [2], [3], [4], [5], [6], [7]. Among them, the 7C of vaccination readiness scale (7C scale) has recently received significant attention [7]. It is a numerical scale that evaluates willingness and readiness to be vaccinated (i.e., vaccination readiness) alongside seven psychological components relevant to vaccination readiness (confidence, complacency, constraints, calculation, collective responsibility, compliance, and conspiracy). Individuals with higher vaccination readiness scores, calculated from the seven psychological components of vaccine readiness, are generally more likely to be vaccinated than those with lower scores. However, it is unclear what the cutoff score should be to determine whether an individual is willing to be vaccinated. When the cutoff score is identified, it is possible to assess the presence or absence of vaccination readiness as a binary category (i.e., high or low). It makes the evaluation of the 7C scale results easier to understand and more useful for promoting public awareness (e.g., revealing the proportion of citizens with high vaccine readiness).

Therefore, this study clarified the cutoff score of vaccination readiness on the 7C scale using receiver operating characteristic (ROC) curve analysis to determine whether an individual is willing to be vaccinated.

## 2 Methods

### 2.1 Study sample and data collection

This cross-sectional study was conducted through an Internet-based survey in Japan. We utilized the datasets collected in a study on the development and validity of the Japanese version of the 7C scale, which comprised 1000 participants [8]. Participants were recruited from among the registrants of a Japanese Internet research service company called iBRIDGE Corporation, which had approximately 4.5 million registered participants at the time of the study. The Internet research service company invited their registered participants to take the survey via their website accounts on March 9, 2022. The detailed sample collection method we used has previously been reported elsewhere [8]. The inclusion criteria for this study were 1) registrants of the Internet research service company, 2) people over 20 years of age, and 3) those who responded to the survey. The exclusion criteria were 1) people who responded in a short time (≤1 min) and 2) those with identical answers to the 21 items of the 7C scale. The method we used is a means through which survey satisficing behavior (e.g., not responding after careful consideration of each item or choosing identical answers) is identified from the data collected in an online survey [9], [10].

This study was approved by the ethics committee of our university in Tokyo, Japan (no: T2021-0265). Informed consent was obtained from all respondents after the nature of the study had been fully explained.

### 2.2 Vaccination readiness score

The 7C scale has a full version with three items for each of the seven questions (a total of 21 questions) and a short version with one item for each of the seven questions (a total of seven questions) [7]. The questionnaire is shown in Supplementary Table 1. In this study, the participants were asked to respond to the Japanese full version of the 7C scale, and the full and short versions were each subjected to separate analyses. Responses to each item were rated on a 7-point Likert-type scale. Each question was coded, with higher scores indicating greater levels of vaccination readiness (score range: 1–7). The total mean score indicates a participant's vaccination readiness score [11].

### 2.3 COVID-19 vaccination status

As the administration of the COVID-19 vaccine booster had begun at the time of the survey, the COVID-19 vaccine was the main topic of discussion among citizens [12], [13], [14]. Therefore, this study established participants’ COVID-19 vaccination status as a criterion for their willingness to be vaccinated. The participants were asked how many times they had received the COVID-19 vaccine. At the time of the survey, approximately 75 % of the people had received the second dose of the COVID-19 vaccination in Japan [13], [14], implying that most people who wanted to be vaccinated had received the second dose. Meanwhile, the booster vaccination coverage was approximately 30 %, and it was rising daily [13], [14], indicating that although some people wanted the booster shot, they had not received it yet at the time of the survey; thus, they were considered as not vaccinated. Therefore, in this study, those who had received more than two doses of vaccination were defined as willing to be vaccinated.

### 2.4 Sociodemographic factors

Each of the participants reported their level of educational attainment. The categorized data provided by the research company comprised information regarding sex, age, and employment status.

### 2.5 Statistical analysis

ROC curve analysis was performed using the Youden Index [15]. The cutoff value of the vaccination readiness score, which determined whether participants were willing to be vaccinated, was clarified. The sensitivity, specificity, and area under the curve (AUC) were reported. Based on previous studies [16], the AUC values were interpreted as excellent (≥0.90), good (0.80–0.89), fair (0.70–0.79), and poor (<0.70).

R version 4.2.0 and “pROC” from the R package were used to perform all statistical analyses.

## 3 Results

In this survey, 1,000 participants responded to the questionnaire. The mean response time to the questionnaire in the survey was 2 min and 40 s. Of the participants, 226 were excluded for the following reasons: response time ≤ 1 min (n = 203), and identical answers were chosen for the 21 items of the 7C scale (n = 23). Therefore, the analysis set comprised 774 participants (Table 1).

**Table 1:** Participants’ characteristics.

	Participants: n = 774			
	n	(%)	mean	(SD)
Sex (men)	296	(38.2 %)		
Age, years			53.2	(16.5)
Educational attainment				
University graduate or above	325	(42.0 %)		
Below university graduate level	449	(58.0 %)		
Employment status				
Working	415	(53.6 %)		
Not working	359	(46.4 %)		
COVID-19 vaccination status, number of vaccine doses				
0–1	125	(16.1 %)		
2–3	649	(83.9 %)		
7C scale				
Vaccination Readiness Score (full version)			4.48	(0.84)
Vaccination Readiness Score (short version)			4.40	(0.95)

*Note.* SD; standard deviation.

**Fig. 1:**
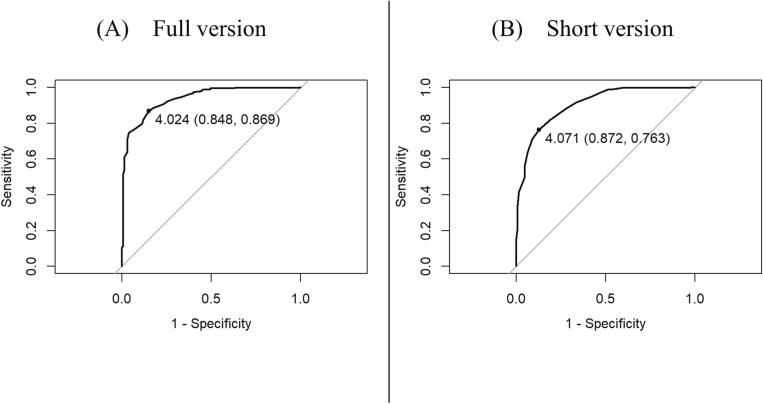
Receiver operating characteristic (ROC) curve analysis for the cutoff value of the vaccination readiness score that determines whether an individual is willing to be vaccinated.

Fig. 1 presents the results of the ROC curve analysis. In the 7C scale full version, when the cutoff value was 4.02, the Youden Index reached the maximum (Youden Index = 0.72, sensitivity = 86.9 %, specificity = 84.8 %, AUC = 0.94) [Fig. 1 (A)]. In the 7C scale short version, when the cutoff value was 4.07, the Youden Index reached its maximum (Youden Index = 0.64, sensitivity = 76.3 %, specificity = 87.2 %, AUC = 0.90) (Fig. 1 (B)).

## 4 Discussion

We set out to clarify the cutoff value of the vaccination readiness score of the 7C scale to determine whether an individual is willing to be vaccinated. ROC curve analysis suggested that the full and short version vaccination readiness scores of 4.02 and 4.07 or higher, respectively, could be used to indicate a willingness to be vaccinated. Considering the range of values that can be obtained for the vaccination readiness score, an individual can be considered willing to be vaccinated if the score is over 4.00 for either the full or short version.

The 7C scale is unique as it can comprehensively assess various psychological factors related to willingness and readiness to be vaccinated. Several recent studies on vaccine hesitancy have used the 7C scale [17], [18], [19], [20]. However, absolute criteria, such as cutoff values, were not clear. This study demonstrated that it is possible to determine cutoff values with acceptable sensitivity and specificity for determining whether the willingness to be vaccinated exists in the vaccination readiness score of the 7C scale.

Some limitations should be considered in our study. First, participants were recruited from a single Internet research company. Therefore, the results may not be generalizable to all populations, such as older adults who do not use the Internet. Second, the COVID-19 vaccination status may have affected the vaccination readiness score, owing to the cross-sectional nature of our study. The experience of receiving a COVID-19 vaccine may increase confidence in it and increase vaccination readiness scores, or vice versa. Further longitudinal studies are required to confirm these effects. Third, our results may not be generalizable. Vaccine hesitancy sometimes varies across places and types of vaccines [21]. The cutoff values of vaccination readiness scores may differ for other vaccines or populations. Despite these limitations, our study provides novel evidence that the 7C scale may provide an acceptable cutoff value for vaccination readiness score for determining whether an individual is willing to be vaccinated. Our results suggest that the 7C scale alone can provide a comprehensive determination of both willingness to be vaccinated and the respondents’ thoughts about vaccination, which makes this scale more convenient in the field.

## 5 Conclusion

On both the full and short versions of the 7C scale, vaccination readiness scores over 4 may indicate a willingness to be vaccinated. Consequently, this cutoff value facilitates the interpretation of the results of the 7C scale and helps when considering education campaigns regarding vaccine hesitancy utilizing the 7C scale.

## Declaration of Competing Interest

The authors declare that they have no known competing financial interests or personal relationships that could have appeared to influence the work reported in this paper.

## Data Availability

The authors do not have permission to share data.
