# Supplementary material for: Estimation of cutoff score for the 7C of vaccination readiness scale
Source: Vaccine X. 2023 Sep 26;15:100394. doi: 10.1016/j.jvacx.2023.100394 (PMC10550591; doi:10.1016/j.jvacx.2023.100394)
Supplement: Supplementary data 1 [file mmc1.docx]

Supplementary Table 1. English and Japanese versions of the 7C scale

| Components  (subscale) | 7C scale English version [7] ^1^ | 7C scale Japanese version [8] ^1^ |
| --- | --- | --- |
| – | Q. The following statements refer to all infectious diseases for which a vaccination is available and recommended by health authorities. Please state how much you agree with each of the statements. Rate the statements from 1 = “strongly disagree” to 7 = “strongly agree”. | 以下の項目は、保健機関が推奨しているワクチンが存在するすべての感染症についての記述です。それぞれの項目にどの程度同意するか、1=「全く同意しない」から7=「強く同意する」の中でお答えください。 |
| Confidence | Vaccination side effects occur rarely and are not severe for me. | ワクチンの副反応はまれにしか起こらず、私にとって深刻ではない。 |
|  | Political decisions about vaccinations are scientifically grounded. | 予防接種に関する政治的判断は、科学的根拠に基づいて行われる。 |
|  | **I am convinced the appropriate authorities do only allow effective and safe vaccines.** ^2^ | **政府関係機関が効果的で安全なワクチンのみを許可すると確信している。** |
| Complacency | I do not need vaccinations because infectious diseases do not hit me hard. (R) ^3^ | 感染症は私に大きな影響を与えないので、予防接種は必要ない。 |
|  | Vaccinations are unnecessary for me because I rarely get ill anyway. (R) | 私はめったに病気にならないので、予防接種は不要だ。 |
|  | **I get vaccinated because it is too risky to get infected.** | **感染すると非常に危険なので、私は予防接種を受ける。** |
| Constraints | I make sure to receive the most important vaccinations in good time. | 私は、最も重要な予防接種を適切な時期に確実に受けるようにする。 |
|  | **Vaccinations are so important to me that I prioritize getting vaccinated over other things.** | **予防接種は私にとってとても大切なので、他のことよりも優先する。** |
|  | I sometimes miss out on vaccinations because vaccination is bothersome. (R) | 予防接種は面倒なので、予防接種の機会を逃すことがある。 |
| Calculation | I get vaccinated when I do not see disadvantages for me. (R) | 私にとってデメリットが見当たらない場合、予防接種を受ける。 |
|  | **I only get vaccinated when the benefits clearly outweigh the risks. (R)** | **利益が危険性より明らかに上回る場合にのみ、予防接種を受ける。** |
|  | For each vaccine, I carefully consider whether I need it. (R) | それぞれのワクチンについて、自分に必要かどうかを慎重に検討する。 |
| Collective responsibility | I also get vaccinated because protecting vulnerable risk groups is important to me. | 感染に弱い人達を守ることも私にとっては重要なので、私は予防接種を受ける。 |
|  | **I see vaccination as a collective task against the spread of diseases.** | **予防接種は病気の蔓延を防ぐための集団行動だと思う。** |
|  | I also get vaccinated because I am thereby protecting other people. | 他の人を守ることができるので私は予防接種を受ける。 |
| Compliance | It should be possible to exclude people from public activities (e.g., concerts) when they are not vaccinated against a specific disease. | 特定の病気の予防接種を受けていない人をイベント（コンサートなど）から排除することが可能になるようにすべきだと思う。 |
|  | The health authorities should use all possible means to achieve high vaccination rates. | 保健機関は、高い接種率に到達するためにあらゆる手段を講じるべきである。 |
|  | **It should be possible to sanction people who do not follow the vaccination recommendations by health authorities.** | **保健機関による予防接種の推奨に従わない人には、制裁を加えることができるようにすべきだ。** |
| Conspiracy | **Vaccinations cause diseases and allergies that are more serious than the diseases they ought to protect from. (R)** | **ワクチンの接種は、それが本来防ぐ病気よりも、もっと深刻な病気やアレルギーを引き起こす。** |
|  | Health authorities knuckle under to the power and influence of pharmaceutical companies. (R) | 保健機関は製薬会社の権力と影響力に屈している。 |
|  | Vaccinations contain chemicals in toxic doses. (R) | 予防接種には、毒性のある化学物質が含まれている。 |

^1^ This study used the 7C scale Japanese version.

^2^ Bold items represent those from the short version of the 7C scale.

^3^ Items with (R) are reverse-coded.
